# Ethanol Sclerosis Therapy for Aggressive Vertebral Hemangioma of the Spine: A Narrative Review

**DOI:** 10.3390/jcm12123926

**Published:** 2023-06-08

**Authors:** Juichi Tonosu, Yasuteru Yamaguchi, Akiro Higashikawa, Kenichi Watanabe

**Affiliations:** Department of Orthopedic Surgery, Kanto Rosai Hospital, Kawasaki 211-8510, Kanagawa, Japan; yasuteruyamaguchi@yahoo.co.jp (Y.Y.); a.higashikawa@gmail.com (A.H.); ken1127watanabe@gmail.com (K.W.)

**Keywords:** hemangioma, spine, vertebra, ethanol, sclerosis

## Abstract

Vertebral hemangiomas of the spine are rare benign tumors. They occur primarily in the thoracic region and are often asymptomatic and found incidentally on radiological examination; however, some are symptomatic, aggressive, and gradually increase in size. Various therapeutic approaches have been proposed for their management. This study aimed to review the therapeutic management, focusing on ethanol sclerosis therapy. The PubMed database was searched from inception to January 2023 using the keywords “hemangioma”, “spine OR vertebra”, and “ethanol”. Twenty studies were retrieved, including two letters. The first report of spinal therapy was published in 1994. Ethanol sclerosis therapy is effective in treating vertebral hemangiomas. It is performed independently or in combination with other techniques, such as vertebroplasty using cement and surgery. The therapy is performed under local or general anesthesia with fluoroscopic or computed tomography guidance. A total of 10–15 mL of ethanol is slowly injected via unilateral or bilateral pedicles. Complications of the therapy include hypotension and arrhythmia during the procedure, paralysis immediately after the procedure, and delayed compression fractures. This review could enable the refinement of knowledge regarding ethanol sclerosis therapy, which is a treatment option that could be adopted.

## 1. Introduction

**Figure 1:**
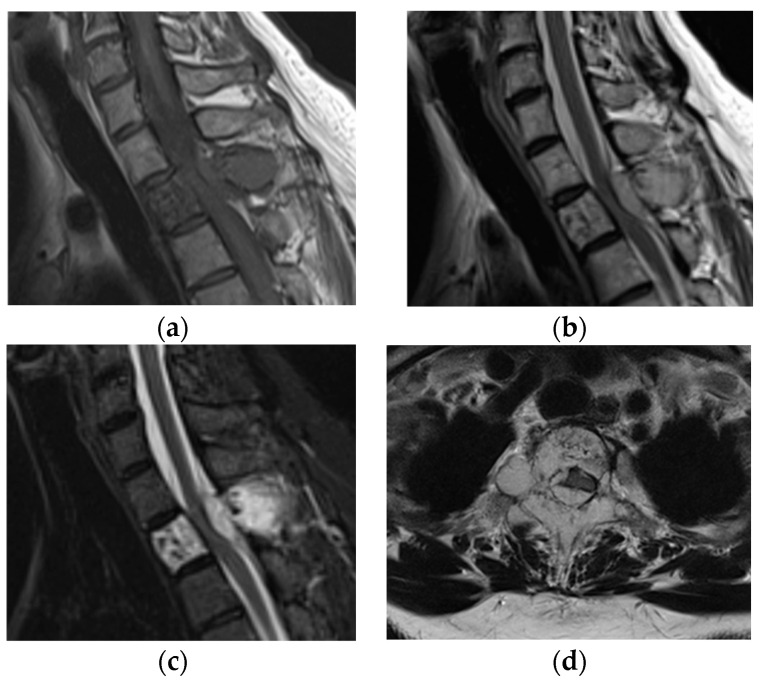
Magnetic resonance imaging of an aggressive vertebral hemangioma of the thoracic spine. A tumor with low intensity on the T1-weighted sagittal image (**a**) and high intensity on the T2-weighted sagittal image (**b**); short tau inversion recovery sagittal image (**c**) and T2-weighted axial image (**d**) compressed the spinal cord. The area had an extra-osseous region on the right.

Vertebral hemangiomas of the spine are rare benign tumors. They are often asymptomatic and found incidentally on radiological examination; however, some are symptomatic, aggressive, and gradually increase in size (Figure 1) [1,2].

The presence of more than three of the following six characteristics is reported to increase the likelihood that a venous hemangioma will be symptomatic: (1) location between T3 and T9; (2) including the entire vertebral body; (3) expanding to the laminas; (4) cortex expansion with an unclear margin; (5) honeycomb pattern; and (6) expanding into the soft tissues [1]. Various therapeutic approaches have been proposed and used to manage symptomatic hemangiomas of the spine [3,4], including vertebroplasty using cement [5,6], radiation therapy [7,8], transarterial embolization [9], and complete tumor resection [10,11]. Ethanol sclerosis therapy was first reported by Heiss et al. in 1994 [12]. In this technique, highly concentrated ethanol is injected into the hemangioma to induce coagulative necrosis in the vascular endothelium and surrounding tissues, followed by irreversible vascular occlusion.

Although there are various treatment options as mentioned above, ethanol sclerosis therapy can be a curative option for the tumor. If ethanol sclerosis therapy has more advantages than disadvantages, it would be a good treatment option. Systematic reviews concerning the management of aggressive vertebral hemangiomas have been published extensively [2,3]. However, reviews focusing on ethanol sclerosis therapy for the spine have not been published. If one is thinking of ethanol sclerosis therapy for a spinal tumor, a refined narrative review would be useful. The study aimed to review therapeutic management, focusing on ethanol sclerosis therapy.

## 2. Materials and Methods

We searched the PubMed database from inception to January 2023, using the keywords “hemangioma”, “spine OR vertebra”, and “ethanol”. Only studies published in English were included in the analysis. We chose the Pubmed database as it is a widely accepted search engine worldwide.

## 3. Results

Twenty studies—including two letters, case series (Table 1), and case reports (Table 2)—were retrieved.

The first report of two cases of ethanol sclerosis therapy for the spine was published by Heiss et al. [12]. They performed the therapy with computed tomography (CT) guidance under local anesthesia and sedation. The approach was unilateral. A 17-gauge bone biopsy needle was used. Ten milliliters of 100% ethanol were injected slowly and intermittently (2 mL of ethanol was injected every 5–10 min). The report recommended the injection of a water-soluble contrast medium before and after ethanol injection. Pre-injection was intended to ensure no leakage into the epidural space and rapid disappearance of the contrast medium owing to the rich vessel flow, whereas post-injection was intended to ensure slow disappearance of the contrast medium with the completion of sclerosis. The first case was of a 54-year-old woman with a T4 hemangioma. She had bilateral lower-limb motor paralysis and could walk only a few steps using a walker. CT-guided transpedicular ethanol sclerosis therapy was performed, followed by unsuccessful transarterial embolization of the 4th intercostal artery. Her motor paralysis and walking ability improved after one week. Magnetic resonance imaging (MRI) obtained ten weeks after the therapy demonstrated that the tumor had not completely shrunk; therefore, additional ethanol sclerosis therapy was performed. The patient was able to walk without assistance within one month. The second case was that of a 64-year-old woman with a T12 hemangioma. She had motor paralysis and bilateral lower limb pain and could walk only six meters. CT-guided transpedicular ethanol sclerosis therapy was performed in a manner that is similar to that described above. Her back pain increased during the ethanol injection; however, paralysis and pain in the lower limbs did not worsen. Her motor paralysis and walking ability improved within two weeks. She was able to walk three kilometers per day within three months.

### 3.1. Case Series 

#### 3.1.1. Case Series of Ethanol Sclerosis Therapy without an Accompanying Procedure

Yadav et al. have published a prospective case series [13]. Eleven patients (seven females and four males) received ethanol sclerosis therapy via a unilateral pedicle using a 20-gauge needle under general anesthesia. Whether fluoroscopy or CT was used for assistance was not reported. The mean age of the participants was 22 years (range: 10–36 years). The hemangioma was located in the thoracic spine in all the cases. All patients developed hypotension and bradycardia at the time of the ethanol injection. The decrease in blood pressure and heart rate persisted until a gradual return to baseline values within 3–4 min. The authors recommend performing the procedure under general anesthesia because of the cardiovascular changes. This study focused on the aforementioned complications during the procedure, and the results and effectiveness of the procedures were not described.

Bas et al. demonstrated that 18 patients (seven females and eleven males) received ethanol sclerosis therapy via unilateral (12 cases) or bilateral (5 cases) pedicles using a 14-gauge bone biopsy needle under local anesthesia with fluoroscopic guidance [14]. The mean age of the participants was 49 years (range: 18–77 years). The hemangioma was located in the thoracic spine in 6 cases and the lumbar spine in 12 cases. All the patients had back pain without paralysis. The mean follow-up duration was 24 months. Sixteen of 18 patients showed excellent clinical results. Two milliliters of contrast medium injection prior to ethanol injection revealed leakage into the spinal canal in the other six cases, in which the subsequent ethanol injection was discontinued. Ethanol was slowly injected. Neurological complications were not observed. Two punctures of the dura mater with cerebrospinal fluid leakage were resolved by changing the position of the needle.

Goyal et al. studied 14 patients (4 females and 10 males), who received ethanol sclerosis therapy via unilateral pedicles using an 18-gauge spinal needle under local anesthesia with CT guidance [15]. The mean age of the participants was 36.9 years (range: 15–59 years). The hemangioma was located in the thoracic spine in eight patients and the lumbar spine in six patients. All patients, except one, experienced paralysis. The follow-up periods were 5–31 months. In total, the clinical results were excellent in 5 cases, good in 8, and poor in 1. Patients were administered oral steroids (prednisone 4–6 mg, 6 h) 48 h prior to the procedure. All patients received 25 mg of pentazocine intramuscularly for analgesia during the procedure. An 8 mL contrast medium injection prior to ethanol administration revealed leakage to the spinal canal in one case, in which the subsequent ethanol injection was canceled. Ethanol was slowly injected. All patients showed transient neurological deterioration in the form of worsening paraparesis that lasted up to 5 days after the procedure. The patients continued receiving oral steroids. Subsequently, 12 patients showed significant improvement. One patient showed significant clinical improvement in the initial 3 weeks but deteriorated rapidly thereafter. Transarterial embolization significantly improved the patient’s condition. Late complications were described as follows: paravertebral abscess in 1 case and vertebral fracture in 3, of which 2 cases were asymptomatic. One patient developed recurrence one month later, followed by laminectomy and transarterial embolization, which she subsequently responded to well.

Doppman et al. studied 11 patients (7 females and 4 males) who received ethanol sclerosis therapy via unilateral pedicles using a 16- or 17-gauge bone biopsy needle under local anesthesia with CT guidance [16]. The mean age of the participants was 51 years (range: 29–73 years). The hemangioma was located in the thoracic spine in seven patients and the lumbar spine in four patients. The mean follow-up period was 40.6 (range: 15–76) months. Symptoms improved within a few days. Five out of six patients with paralysis completely recovered walking without support, whereas one partially recovered. Of the five cases of radiculopathy, three recovered partially and two recovered completely. No early complications occurred in any of the patients. Two patients showed delayed pathological vertebral fractures 4 and 16 weeks after the procedure, respectively. The volume of ethanol in these two cases was 42 and 50 mL, respectively. The authors suggested that a large amount of ethanol could lead to vertebral fractures and recommended the use of <15 mL.

#### 3.1.2. Case Series of Ethanol Sclerosis Therapy Accompanied by Vertebroplasty 

Premat et al. studied 26 patients (15 females and 11 males), who received ethanol sclerosis therapy followed by percutaneous vertebroplasty 2 weeks after ethanol injection [17]. Ethanol injection was performed via unilateral (4 cases) or bilateral pedicles (22 cases) using a 13-gauge needle under general anesthesia (18 cases) or sedation (8 cases) with fluoroscopic guidance. The mean age of the participants was 51.8 years (range: 19–75 years). The hemangioma was located in the thoracic spine in 14 patients and the lumbar spine in 13 patients. The mean follow-up period was 88.3 (range: 22–217) months. The mean visual analog scale (VAS) score of back pain changed from 7.23 to 3.11 after the procedure. Two of three patients with preoperative motor paralysis did not recover. Eight of nine patients with sensory symptoms recovered completely. No major complications occurred during the postoperative or follow-up periods.

Chen et al. reported a retrospective case series of 12 patients (7 females and 5 males), who received ethanol sclerosis therapy and vertebroplasty on the same day [18]. After vertebroplasty using cement, ethanol was injected via a unilateral pedicle (the opposite side for cement vertebroplasty) using a 14-gauge bone biopsy needle under local anesthesia with fluoroscopic guidance. The mean age was 41 years (range: 26–54 years). The hemangioma was located in the thoracic spine in 6 cases and the lumbar spine in 6 cases. The mean follow-up duration was 29 months. The symptoms were described as back pain. However, motor and sensory disturbances were not described. Eleven of the 12 patients showed significant improvement in back pain. The mean VAS score changed from 6.5 to 1.7. There were no early complications associated with ethanol injection. There were no recurrences of the tumor or vertebral fractures. A vertebral fracture at the adjacent upper level was observed at the one-year follow-up in one case.

#### 3.1.3. Case Series of Ethanol Sclerosis Therapy Accompanied by Surgery

Chandra et al. reported 33 cases (18 females and 15 males) in 2019 [19]. The cases were those of spinal hemangiomas with myelopathy that were followed for more than two years. Following transarterial embolization, posterior decompression, and fixation surgeries with ethanol injections were performed. After pedicle screws were placed at the adjacent level above and below the healthy vertebrae, two 14–16-gauge Jamshidi needles were inserted into the affected vertebra with fluoroscopic guidance. The hemangioma was located in the thoracic spine in 32 patients and the lumbar spine in 1 patient. The mean age of the participants was 26.9 years (range: 10–68 years). Ethanol was injected slowly, with each bolus not exceeding 0.5 mL. The mean total time of surgery and blood loss were 124 min and 274 mL, respectively. The mean follow-up period was 47.6 (range: 28–103) months. Postoperative imaging revealed sclerosis of the vertebral body and an improvement in spinal cord compression in all cases. All cases, except one, showed improvement in paralysis. The preoperative American Spinal Injury Association (ASIA) grade was as follows: A, 7 cases; B, 11 cases; C, 6 cases; D, 8 cases; and E, 1 case. The postoperative ASIA grade was as follows: A, 0 cases; B, 2 cases; C, 1 case; D, 4 cases; and E, 26 cases. Complications occurred in two cases. One was postoperative deterioration in power in both lower limbs, which improved within two weeks. Other causes were transient intraoperative hypotension and arrhythmia.

Singh et al. reported 7 cases (5 females and 2 males) in 2015. The patients received an ethanol injection via bilateral pedicles under fluoroscopic guidance, followed by posterior decompression and fixation surgery [20]. The mean age of the participants was 14 years (range: 10–17 years). The hemangioma was located in the thoracic spine in all the cases. The mean total time of surgery and blood loss was 248.6 min and 535 mL, respectively. The mean follow-up period was 45.3 (range: 1–78) months. All the patients showed motor and sensory improvements. The operative procedure and complications were similar to those in the aforementioned case series [19]. Considering the authors’ names and affiliations, the cases in these two studies may have overlapped.

### 3.2. Case Reports

Some case reports have shown good results. Chen et al. demonstrated that a 27-year-old woman with an L3 hemangioma presented with sudden onset of lower limb pain and incomplete paralysis below the hip flexors [21]. Ethanol sclerosis therapy was administered three days after transarterial embolization. The procedure was performed via a unilateral pedicle using a bone biopsy needle under local anesthesia with CT guidance. Ethanol was then injected. Her paralysis resolved immediately after the therapy. Partial collapse of the vertebral body was observed nine months after the procedure, with minimal pain.

Degulmadi et al. demonstrated that a 56-year-old woman with hemangiomas of multiple vertebral bodies (T2, T4, T9, T12, L3, and L5) presented with back pain, incomplete paralysis of the bilateral lower limbs, and bladder dysfunction [22]. The vertebral body that compressed the dura mater was T12. Ethanol sclerosis therapy was performed at T12 under local anesthesia using fluoroscopy. The procedure was performed via a unilateral pedicle using a 16-gauge Jamshedi needle under local anesthesia and fluoroscopic guidance. Ethanol was slowly injected. Second, posterior decompression and fixation surgery from T7 to L4, along with transpedicular curettage and transplantation of the iliac bone to T12, were performed under general anesthesia 24 h after ethanol injection. The volume of blood loss was 450 mL, which was regarded as low as estimated owing to the initial sclerosis therapy. Her paralysis began to improve four days after surgery. Motor power improved after three weeks. The bladder recovered within two weeks. At the one-year follow-up, the patient reported an almost complete absence of back pain.

Cianfoni et al. reported that a 78-year-old woman with T8 and T9 hemangiomas presented with incomplete paralysis of both lower limbs [23]. Ethanol sclerosis was performed via bilateral pedicles using 13-gauge vertebroplasty needles under general anesthesia with CT guidance. Ethanol was injected slowly. It was performed first at T8 and two days later, at T9. His paralysis resolved a few hours after therapy.

Niemeyer et al. reported a case of Brown–Sequard syndrome as a complication following therapy [24]. The patient was a 27-year-old man with a T8 hemangioma who did not develop neurological disturbances. Ethanol sclerosis was performed via a unilateral pedicle using a 17-gauge biopsy needle under local anesthesia with fluoroscopic guidance. Ethanol was slowly injected. After 7 mL of ethanol was injected, the procedure was terminated because of unbearable back pain. Incomplete motor paralysis on the right side and sensory disturbance on the left side developed one hour after therapy. Bladder function was not impaired. Methylprednisolone was intravenously administered. Four hours after therapy, the patient’s motor paralysis started to improve at two to three levels in manual muscle testing, although the sensory disturbance remained unchanged. Emergency decompression and fixation surgery revealed marked thrombosis of the epidural veins on the entry side. His motor paralysis recovered completely within six weeks although a slight sensory disturbance persisted. The authors suspected that thrombosis of the epidural veins led to spinal cord ischemia. The authors mentioned that back pain during the injection should be considered.

## 4. Discussion

Vertebral hemangiomas of the spine occur mainly in the thoracic vertebrae and sometimes in the lumbar vertebrae. Ethanol sclerosis therapy is effective in treating vertebral hemangiomas. It was performed independently or in combination with other techniques, such as vertebroplasty using cement and surgery. Most studies were performed with reference to the first report by Heiss et al. [12]. Therapy was performed under local or general anesthesia with fluoroscopic or CT guidance. A total of 10–15 mL of ethanol was slowly injected via unilateral or bilateral pedicles using 13–20-gauge needles. Complications of the therapy include hypotension and arrhythmia during the procedure, paralysis immediately after the procedure, and delayed compression fractures. 

Hypotension and arrhythmia could be related to the effect of ethanol on the systemic circulation. Although the importance of careful monitoring for cardiovascular changes has been mentioned, preventive measures have not yet been discussed. Yadav et al. demonstrated that the decrease in blood pressure and heart rate gradually returned to baseline values within 3–4 min [13]. If the event is reversible and short-lived, the preparation of intravenous drugs for cardiovascular changes during the procedure would resolve the problem.

Both the total volume and injection speed of ethanol may be related to paralysis. The appropriate volume and speed were considered in the report by Heiss et al. [12]. They could differ by the volume of vertebral bodies, which means that the volumes for the upper thoracic and lumbar vertebrae could be different. However, this has not been investigated in previous studies. Slow injection prevented rapid sclerosis of the vertebral bodies, which is related to thrombosis of the epidural veins, and also prevented the vigorous seeping of ethanol through the fragile bony wall into the epidural space. However, this mechanism has only been proposed as a hypothesis. Unbearable back pain during ethanol injection can indicate the clinical progression of paralysis [24]. From this point of view, therapy under local anesthesia could be recommended. If the therapy is performed under general anesthesia, transcranial motor-evoked potential monitoring of neuromuscular function would be effective in preventing paralysis. Goyal et al. intended to prevent paralysis by administering steroids to patients before and after the procedure [15]. However, all patients showed transient neurological deterioration, indicating that steroids might not be effective at preventing paralysis. Although paralysis is transient, paralysis prevention methods are expected to be developed in the future.

Delayed compression fracture could be related to fragility due to hypovascular vertebral bodies after ethanol injection. The reason the fractures were often asymptomatic could also be related to coagulative necrosis of the vertebral bodies. Additional vertebroplasty using cement or posterior fixation surgery has been considered to overcome this fragility. A case series of ethanol sclerosis therapy accompanied by vertebroplasty demonstrated no recurrence and no intraoperative complications during the follow-up periods of 29 and 88.3 months, respectively [17,18]. However, there is a potential risk of cement leakage into the spinal canal through the fragile posterior wall of the vertebral body. In addition, although the recurrence rate might be low, surgical resection may be difficult after recurrence due to the presence of cement. From the viewpoint of a curative option for the tumor, entire tumor resection as total en block spondylectomy (TES) is the best technique. However, it may be difficult due to bleeding from a hypervascular tumor. A case series demonstrated various types of surgeries for the tumor and concluded that only TES had shown poor results due to some complications [25]. Degulmadi et al. indicated a good alternative strategy in their case report [22]. The posterior decompression and fixation surgery along with transpedicular curettage and transplantation of the iliac bone was performed one day after the ethanol sclerosis therapy, which decreased the blood loss. Curettage could be acceptable because hemangioma is a benign tumor. On the contrary, the results of two case series with surgery indicated that curettage of the tumor is not mandatory [19,20]. During their follow-up periods of 45.3 and 47.6 months, respectively, neither recurrence nor delayed compression fractures were described. In summary, posterior decompression and fixation surgeries with ethanol injections could be a preferable strategy for preventing delayed compression fractures.

A limitation was that we only searched the PubMed database.

## 5. Conclusions

We retrospectively reviewed the therapies for aggressive vertebral hemangiomas of the spine, focusing on ethanol sclerosis therapy. This review could enable us to refine our knowledge of the therapy. Ethanol sclerosis therapy is a treatment option that could be adopted; however, there are some unresolved complications such as intraprocedural cardiovascular change, the possibility of paralysis, and late vertebral collapse. The outcomes of stand-alone ethanol sclerosis therapy are inferior to those of ethanol sclerosis therapy combined with other techniques. It is important to reduce complications associated with therapy.

## 6. Future Directions

Future studies are also expected to reveal the pathological mechanism of injection-related paralysis.

## Figures and Tables

**Table 1:** Details of the case series on ethanol sclerosis therapy.

No.	Authors	Number of Cases	Mean Age	Female/Male	Mean Follow-Up Period [Range] (Months)	Mean Amount of Ethanol [Range] (mL)	Accompanied Procedure
1	Heiss et al. [12]	2	59	2/0	5.5	10	Transarterial embolization/None
2	Yadav N et al. [13]	11	22	7/4	Not provided	[8–10]	None
3	Bas et al. [14]	18	49	7/11	24	10	None
4	Goyal M et al. [15]	14	36.9	4/10	[5–31]	[10–15]	None
5	Doppman JL et al. [16]	11	51	7/4	40.6	16.5	None
6	Premat K et al. [17]	26	51.8	15/11	88.3	5.14	Vertebroplasty
7	Chen L et al. [18]	12	41	7/5	29	5	Vertebroplasty
8	Chandra et al. [19]	33	26.9	18/15	47.6	14.6	Transarterial embolization Posterior decompression and fixation surgery
9	Singh PK et al. [20]	7	14	5/2	45.3	12.8	Transarterial embolization Posterior decompression and fixation surgery

**Table 2:** Details of case reports on ethanol sclerosis therapy.

No.	Authors	Age	Sex	Location	Amount of Ethanol (mL)	Injection Speed (mL/min.)	Accompanied Procedure	Result
1	Chen HI et al. [21]	27	F	L3	9	Not provided	Transarterial embolization	Immediate improvement of paralysis
2	Degulmadi D et al. [22]	56	F	T12	8	0.5	Posterior decompression and fixation surgery with transpedicular curettage and bone transplantation	Improvement of paralysis began 4 days after the therapy
3	Cianfoni A et al. [23]	78	M	T8, T9	10 (each)	0.2	None	Immediate improvement of paralysis
4	Niemeyer T et al. [24]	27	M	T8	7	0.2	None	Development of incomplete paralysis 1 h after the therapy

## Data Availability

The data supporting the conclusions of this article will be shared on the PubMed database.
